# Epidemiology and Clinical Characteristics of Laboratory-Confirmed COVID-19 and Influenza Infections in Children: A 2015–2024 Study in Taiwan

**DOI:** 10.3390/microorganisms13030517

**Published:** 2025-02-26

**Authors:** Hao-Yuan Lee, Chien-Chin Chen, Shu-Hua Ko, Yu-Lung Hsu, En-Pen Chang, Yu-Chau Hsu, Meng-Yen Li, Chyi-Liang Chen, Wen-Yuan Lee

**Affiliations:** 1 Department of Nursing, Jen-Teh Junior College of Medicine, Nursing and Management, Miaoli 35664, Taiwan; d9700101@gmail.com; 2 Department of Pediatrics, Wei Gong Memorial Hospital, Miaoli 35159, Taiwan; 045198@tool.caaumed.org.tw (C.-C.C.); 045652@tool.caaumed.org.tw (S.-H.K.); 045268@tool.caaumed.org.tw (E.-P.C.); 010785@tool.caaumed.org.tw (Y.-C.H.); 045441@tool.caaumed.org.tw (M.-Y.L.); 3 School of Medicine, College of Medicine, Fu Jen Catholic University, New Taipei 242062, Taiwan; 4 Division of Pediatric Infectious Diseases, China Medical University Children’s Hospital, China Medical University, Taichung 40447, Taiwan; dan5230@gmail.com; 5 Molecular Infectious Disease Research Center, Chang Gung Memorial Hospital at Linkou, Taoyuan 333423, Taiwan; 6 Department of Microbiology and Immunology, College of Medicine, Chang Gung University, Taoyuan 33302, Taiwan; 7 Department of Neurosurgery, China Medical University Children’s Hospital, China Medical University, Taichung 40447, Taiwan; 8 Department of Neurosurgery, Wei Gong Memorial Hospital, Miaoli 35159, Taiwan

**Keywords:** influenza, COVID-19, Omicron BA.2.3.7, Omicron BA.5, Omicron JN.1, epidemiology, clinical presentations

## Abstract

The COVID-19 pandemic and Taiwan’s strict prevention policies from May 2020 to April 2023 significantly altered the epidemiology of viral infections. This study analyzed pediatric COVID-19 and influenza cases at Wei Gong Memorial Hospital from January 2015 to March 2024. Clinical features were compared among children hospitalized during the Omicron BA.2.3.7 (April–July 2022), Omicron BA.5 (August–December 2022), and Omicron JN.1 (2024) waves and those with influenza in 2024 and 2015–2016. Between 2015 and 2024, there were 2729 influenza A (36.6%), 974 influenza B (13.1%), and 3752 COVID-19 (50.3%) cases, with 84.7% of COVID-19 cases occurring in May–December 2022. In 2024, high fever (>40 °C) was more common in influenza A cases (32.9%) than in COVID-19 or influenza B (*p* < 0.004). Leukocytosis (>12,000/µL) was more frequent in COVID-19 cases (33.3%) than in influenza A (12.2%) or B (0%) (*p* < 0.001). Pneumonia was more prevalent in COVID-19 cases in 2024 (27.3%) than in 2022 (*p* ≤ 0.030), and pneumonia rates for influenza A (14.8%) and B (16.7%) in 2024 exceeded those in 2015–2016 (*p* ≤ 0.030). Increased pneumonia rates in 2024 emphasize the importance of vigilance and timely intervention for pediatric COVID-19 and influenza cases.

## 1. Introduction

The COVID-19 pandemic has resulted in over 776 million confirmed cases and more than seven million deaths globally as of 15 September 2024 [1]. Taiwan’s strict prevention measures from May 2020 to April 2023, including universal masking, contact tracing, and social distancing, significantly altered the epidemiology and clinical presentation of viral infections, particularly in children [2]. During this period, influenza cases were nearly absent, but a significant resurgence occurred in 2023, along with a rise in pneumonia cases in 2024, based on our clinical observations.

In 2024, we observed that the number of monthly cases of children infected with COVID-19 and influenza increased simultaneously. We have entered an era where children presenting with fever and respiratory symptoms may be infected with either COVID-19 or influenza. Therefore, understanding the clinical presentations of these two infections is crucial, as they require different treatments.

Several studies have compared the clinical features of children with COVID-19 and influenza [3,4,5,6,7,8,9,10,11,12,13,14,15]; however, many have reported conflicting results or failed to establish clear distinctions. For example, although studies in the United States and China reported median ages of children with influenza around 3.9 years and 27.3 to 37.0 months, respectively [3,16], epidemiological studies in Taiwan, Mexico, and Korea have shown that most children with COVID-19 were aged ≤6 years [4,5,17], whereas studies in the US reported a median age of 8.3 years. A 2021 study in Turkey compared laboratory findings between children with COVID-19 and those with influenza [5]. The study found no significant differences in white blood cell (WBC) counts, neutrophil counts, lymphocyte counts, incidence of neutropenia, hemoglobin levels, platelet counts, C-reactive protein (CRP) levels, aspartate transaminase (AST), or alanine transaminase (ALT) between 71 children with COVID-19 and 74 with influenza before 2021. Similarly, a meta-analysis of studies published between 2019 and 2022 found no significant difference in CRP levels between children with influenza and those with COVID-19 [15].

Data comparing the clinical presentations of COVID-19 and influenza in 2024 remain limited. Additionally, comparisons between these two infections in 2024 and the pre-pandemic period are lacking. To address this gap, we conducted a study examining patients with influenza and SARS-CoV-2 from 2015 to 2024, focusing on epidemiology and clinical presentations.

The Alpha variant of SARS-CoV-2 was first identified in Taiwan in January 2021, followed by the Delta variant, which circulated from June to December 2021 [18]. The Omicron variant emerged in January 2022, with BA.2.3.7 predominating from April to July 2022 [19]. From August to December 2022, BA.5 replaced BA.2, accounting for 54.5% to 100% of sequenced cases. Since January 2024, the JN.1 variant, derived from BA.2.86, has been dominant, comprising ≥77% of cases [20,21]. Distinct clinical presentations and outcomes have been linked to different variants of concern (VOCs) of COVID-19 and may also correlate with the high variability of influenza viruses [22,23]. This study compares the clinical features of children hospitalized during the Omicron BA.2.3.7 (April–July 2022), BA.5 (August–December 2022), and JN.1 (2024) waves, as well as influenza cases from 2015 to 2016 and 2024. Specifically, we examined differences among children with influenza A, influenza B, and COVID-19 during the same period in 2024.

Since pneumonia is the leading cause of severe illness and mortality in young children globally [24,25], this study also focused on cases of both radiologically and clinically confirmed pneumonia complicated by influenza or COVID-19 [24,25,26]. This study also evaluated factors influencing pediatric mortality risk, including the Pediatric Sequential Organ Failure Assessment (p-SOFA) and Pediatric Risk of Mortality III (PRISM III) scores [27,28]. Factors associated with increased scores, such as high fever (>40 °C) and low platelet counts (<200,000/µL), were analyzed.

COVID-19 and its variants significantly impacted the epidemiology of other viruses. For example, influenza virus circulation in the United States sharply declined within two weeks of the COVID-19 emergency declaration in March 2020, with similar trends observed in Denmark, Taiwan, Hong Kong, and Singapore [29,30,31,32]. These changes may have affected the clinical courses of viral infections, necessitating new treatment strategies. Identifying the epidemiology of the most clinically relevant respiratory viruses will aid the development of models of infection and allow for the development of targeted treatments, particularly for populations most vulnerable to respiratory-virus-induced diseases [33]. Therefore, this study aimed to provide valuable insights for the early diagnosis and appropriate treatment of these viral infections in children.

## 2. Materials and Methods

### 2.1. Study Design

The epidemiology and clinical presentations of children with laboratory-confirmed COVID-19 and influenza were analyzed at Wei Gong Memorial Hospital (WGMH), an 828-bed China Medical University Strategic Alliance hospital in Northern Taiwan, from January 2015 to March 2024. Patients with co-infections or incomplete data were excluded. Additionally, patients without national health insurance were excluded to minimize biases related to socioeconomic status or access to healthcare. We analyzed the clinical presentations of children hospitalized with COVID-19 during the Omicron BA.2.3.7 wave (April–July 2022), the Omicron BA.5 wave (August–December 2022), and the Omicron JN.1 wave in 2024. Additionally, we compared these findings to children with influenza infections from 2015 to 2016 and in 2024, focusing specifically on differences among children with influenza A, influenza B, and COVID-19 during the same period in 2024.

COVID-19 was diagnosed via RT-PCR (Xpert^®^ Xpress CoV-2 Plus, Sunnyvale, CA, USA) or the Panbio™ COVID-19 Ag Rapid Test (Abbott Diagnostic GmbH, Jena, Germany). The Xpert^®^ test detects SARS-CoV-2 nucleic acids targeting the N, E, and RdRP genes with sensitivities of 403, 200, and 70 copies/mL, respectively. Nasopharyngeal swabs were processed on a GeneXpert^®^ Dx instrument (Sunnyvale, CA, USA) with internal controls for accuracy [34,35].

The Panbio™ COVID-19 Ag Test (Abbott, Germany) used nasal and oral specimens, with results read within 15 min by trained technicians. Leftover samples (~120 μL) were stored at 4 °C. The test strip contains immobilized anti-SARS-CoV-2 antibodies on the test line and monoclonal anti-chicken IgY on the control line. A positive result is indicated by a test line, while a clear control line ensures validity [36].

Influenza rapid testing was conducted using a Bioline™ Influenza Ultra (Abbott Diagnostics Korea Inc., Yongin-si, Gyeonggi-do, Korea), an immunochromatographic assay designed to detect Influenza A and B antigens from nasopharyngeal swabs or aspirates. The test uses immobilized mouse monoclonal anti-influenza A and B antibodies and shows sensitivities of 88.5% for influenza A and 91.5% for influenza B, with an overall specificity of 97.4% compared to culture methods [37]. Anemia was defined based on the World Health Organization (WHO) criteria according to the patient’s age [38]. Pneumonia was identified using the WHO radiological criteria for confirmed pneumonia and met the WHO clinical case definition, which includes symptoms such as cough or difficulty breathing.

### 2.2. Ethical Statements

This study was approved by the Institutional Review Board of China Medical University Hospital, Taiwan (IRB No. CMUH113-REC1-086).

### 2.3. Statistical Analysis

Pearson’s chi-squared test or Fisher’s exact test was used to compare categorical variables. Continuous variables were analyzed using logistic regression or the Wilcoxon rank-sum test. Variables with a *p*-value < 0.2 in the univariate analysis were included in a forward stepwise manner to develop the final model for the multivariate analysis. All statistical tests were two-sided, with statistical significance set at *p* < 0.05. For normality testing, the Shapiro–Wilk test was applied to small sample sizes (<50), while the Kolmogorov–Smirnov test was used for larger samples (*n* ≥ 50). In both tests, the null hypothesis assumes that the data follow a normal distribution. If *p* > 0.05, the null hypothesis is accepted, indicating a normal distribution [39].

## 3. Results

### 3.1. Epidemiology of Children with Influenza A, Influenza B, or COVID-19 from 2015 to 2024

**Figure 1:**
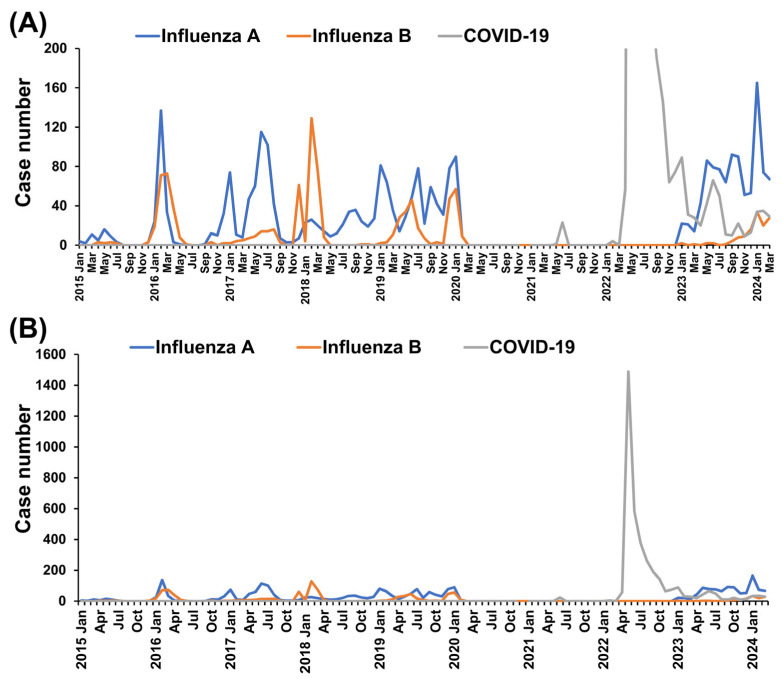
Monthly case numbers of laboratory-confirmed influenza A, influenza B, and COVID-19 among children from 2015 to 2024. (**A**) Monthly case numbers with a Y-axis scale set to a maximum of 1600 to highlight the peak cases during the COVID-19 pandemic. (**B**) Monthly case numbers with a Y-axis scale set to a maximum of 200 to emphasize fluctuations in influenza A and B cases across the study period.

Between 2015 and 2024, there were 2729 recorded cases of influenza A, 974 cases of influenza B, and 3752 cases of COVID-19. Notably, 84.7% of the COVID-19 cases occurred between May and December 2022. The highest number of influenza A cases in a single month was recorded in January 2024 (*n* = 165), followed by February 2016 (*n* = 137). For influenza B, the highest monthly case number occurred in February 2018 (*n* = 129), with the second-highest in March 2016 (*n* = 73) (Figure 1).

From March 2020 to December 2022, no cases of influenza were identified, coinciding with the COVID-19 pandemic in children. The peak monthly case number for COVID-19 occurred in May 2022 (*n* = 1489), followed by June 2022 (*n* = 578) (Figure 1).

### 3.2. Demographic Characteristics of Children with Laboratory-Confirmed SARS-CoV-2 or Influenza Infections

In 2024, a higher percentage of children admitted with COVID-19 were male (69.7%) compared to April–July 2022 (43.8%, *p* = 0.007) (Table 1). The mean age of children with influenza A or B was significantly higher than those with COVID-19 across all three periods (*p* ≤ 0.004). A greater proportion of children under 1 year-old were admitted with COVID-19 in 2024 (and during April–July and August–December 2022) compared to those with influenza A or B (all >19% vs. <7%, *p* < 0.04). Similarly, over 43% of children with COVID-19 in 2024 (and both 2022 periods) were under 3 years old, compared to less than 7% of those with influenza A or B in 2024 or 2015–2016 (*p* < 0.04). In contrast, more children with influenza A or B in 2024, or influenza A in 2015–2016, were aged 6–11 years (>46%) compared to those with COVID-19 in 2024 or August–December 2022 (<20%, *p* < 0.002). Similarly, more children with influenza B in 2024 or 2015–2016 were aged 6–11 years (>33%) than those with COVID-19 across all periods (<16%, *p* < 0.045) (Table 1).

### 3.3. Symptom Characteristics of COVID-19 and Influenza in Children

In 2024, children with influenza A had a significantly higher rate of high fever (>40 °C, 40%) compared to influenza B (3.3%) or COVID-19 (9.1%, *p* < 0.001). This rate was also higher than influenza B in 2015–2016 (14.5%), COVID-19 from April–July 2022 (1.3%), August–December 2022 (5.3%), and influenza A in 2015–2016 (20.2%; all *p* < 0.001). Additionally, 80.8% of children with influenza A had a fever > 39.4 °C, exceeding rates for COVID-19 in 2024 (30.3%), influenza A in 2015–2016 (54.8%), COVID-19 from April to July 2022 (26.1%), August to December 2022 (39.5%), and influenza B in 2024 (53.3%; all *p* < 0.001) (Table 2).

In 2024, children with influenza A or B had a higher incidence of fever lasting ≥3 days (both >75%) compared to COVID-19 in 2024 (39.4%) and August–December 2022 (30.7%; *p* < 0.001). Influenza A in 2024 and 2015–2016, as well as influenza B in 2024, was also associated with more cases of fever lasting ≥4 days (all >45%) compared to COVID-19 in 2024 (18.2%), April–July 2022 (13.1%), and August–December 2022 (19.7%; all *p* < 0.005). Children with influenza B in 2024 had a higher rate of fever lasting ≥4 days (66.7%) than those with influenza B in 2015–2016 (34.9%; *p* = 0.003) (Table 2).

Children with influenza A in 2024, influenza A in 2015–2016, and influenza B (both years) had higher rates of fever lasting ≥5 days (all >25%) compared to COVID-19 in 2024, April–July 2022, and August–December 2022 (all <6%; *p* < 0.001) (Table 2).

The most common symptoms among children with influenza A, B, or COVID-19 across all periods were fever (>94%), cough (>82%), and rhinorrhea (>47%). Children with influenza A in 2015–2016 and 2024 were more likely to experience cough than those with COVID-19 (*p* < 0.009). Similarly, children with influenza B in 2015–2016 were more likely to have a cough than those with COVID-19 from April to July 2022 (Table 2).

Body aches were more frequent in children with influenza A in 2015–2016 (45.2%) compared to those with influenza A in 2024, influenza B in both periods, or COVID-19 across all periods (all <26%, *p* < 0.028). Additionally, children with influenza A or B in both years had a higher incidence of body aches (>16%) compared to those with COVID-19 during the 2022 waves (1.3%, *p* < 0.007). However, children with influenza A or B in 2024 did not show significantly higher rates of body aches (>16%) compared to those with COVID-19 in 2024 (9.1%, *p* > 0.179) (Table 2).

### 3.4. Laboratory Findings in Children with COVID-19 or Influenza

In 2024, 33.3% of children with COVID-19 exhibited leukocytosis (>12,000/μL), significantly higher than those with COVID-19 in 2022 or influenza A/B in both 2024 and 2015–2016 (<13%, *p* < 0.007). Children with influenza B in 2015–2016 had the highest rate of leukopenia (<4000/μL) at 20.5%, compared to influenza A in 2024 or 2015–2016 and COVID-19 in 2024 or 2022 (<9.8%, *p* < 0.029) (Table 3).

Neutrophilia (>7500/μL) was more frequent in children with influenza A in 2015–2016 (25%) than in 2024 (*p* = 0.049). Lymphocytopenia (<2000/μL) was more prevalent in children with influenza A/B in both periods and COVID-19 from April to July 2022 (>73%) than in COVID-19 cases from 2024 or August–December 2022 (<56%, *p* < 0.016) (Table 3).

Lymphocytopenia (<1500/μL) was also higher in children with influenza A/B in both periods (>65%) compared to COVID-19 cases in 2024 or August–December 2022 (<46.2%, *p* < 0.016). In 2024, children with influenza A or B had more lymphocytopenia (<1000/μL) than in 2015–2016 (*p* < 0.001). Similarly, 52.9% of children with COVID-19 from April to July 2022 had lymphocytopenia (<1000/μL), significantly higher than in COVID-19 cases from 2024 or August–December 2022 (≤25%, *p* ≤ 0.001) (Table 3).

In 2024, anemia was more common in children with influenza A (14.8%) than in 2015–2016 (4.8%, *p* = 0.014). Children with COVID-19 from April to July 2022 also had higher anemia rates (15.7%) compared to August–December 2022 (5.3%, *p* = 0.031). Children with influenza B in 2024 had more cases of platelet counts < 200,000/μL (23.3%) compared to influenza A or COVID-19 (both ≤9.6%, *p* > 0.036). Influenza B in 2015–2016 showed an even higher prevalence (41.0%) than influenza B in 2024, influenza A in both years, or COVID-19 across all periods (all ≤25%, *p* ≤ 0.021). Additionally, influenza A in 2015–2016 had more cases of platelet counts < 200,000/μL (25.0%) compared to 2024 (9.6%, *p* = 0.002). COVID-19 from April to July 2022 also had higher rates (26.8%) than August–December 2022 (6.1%, *p* = 0.011) (Table 3).

Children with influenza A in 2024 and 2015–2016 had higher rates of CRP > 1 mg/dL (both > 50%) compared to influenza B in 2015–2016 or COVID-19 (≤32.5%, *p* < 0.043). They also had higher rates of CRP >3 mg/dL (both >14%) than those with influenza B in 2015–2016 or COVID-19 (≤6.1%, *p* < 0.043). Children with COVID-19 from both 2022 periods had higher rates of AST > 38 U/L (both >44%) than those in 2024 (21.2%, *p* = 0.048), while children with COVID-19 in 2024 had higher rates than those with influenza A or B (all ≤4.3%, *p* < 0.004) (Table 3).

### 3.5. Pneumonia Complications in Children with COVID-19 or Influenza

Children with COVID-19 in 2024 had a higher rate of pneumonia (27.3%) compared to the 2022 waves (*p* = 0.030, 0.002). Influenza A in 2024 also had a higher pneumonia rate (14.8%) than in 2015–2016 (4.0%, *p* = 0.006), and influenza B in 2024 had a higher rate (16.7%) than in 2015–2016 (3.6%, *p* = 0.030) (Table 3).

## 4. Discussion

To our knowledge, this is the first comprehensive study comparing children hospitalized with COVID-19 during the Omicron BA.2.3.7 wave (April–July 2022), the Omicron BA.5 wave (August–December 2022), and the Omicron JN.1 wave in 2024, as well as those hospitalized with influenza in 2024 and 2015–2016.

Taiwan had a relatively low incidence of COVID-19 before 2022 [40], with only 24 pediatric cases diagnosed at our hospital. However, from April 2022 to March 2023, the country experienced a nationwide outbreak in three waves [40]. The first two waves (April–August 2022 and August–December 2022) were compared with the 2024 wave. Children with influenza A or B were present from 2015 to February 2020 but disappeared from March to December 2020 due to COVID-19 mitigation measures [41]. With the relaxation of these interventions, influenza resurfaced, reaching a peak in January 2024, surpassing pre-pandemic levels due to “immune debt” [41]. This finding aligns with a study from Denmark that investigated the impact of COVID-19 interventions on the epidemiology of pediatric respiratory infections, demonstrating a decline in incidence during lockdowns followed by a resurgence [29].

In our study, a higher proportion of children admitted with COVID-19 in 2024 and during the two earlier waves (April–July 2022 and August–December 2022) were under 1 year old (19.7–26.1%) or under 3 years old (43.8–50.0%) compared to those with influenza A or B in 2024 or 2015–2016. Similarly, a study conducted at Chang Gung Memorial Hospital (CGMH) in Taiwan reported that approximately 20% of children with COVID-19 were under 1 year old, and more than half were under 6 years old [17]. In contrast, in our study, nearly half of the children with influenza A in 2015–2016 and 2024, as well as those with influenza B in 2024, were aged 6–11 years (46.7–50.8%). A study in China found that the median ages of children infected with influenza were 27.3 months in 2018–2019 and 37.0 months in 2020–2021, both younger than those observed in our study (9–11 years, as shown in Table 1) [16]. Additionally, a study in the US comparing children with COVID-19 in 2020 to those with influenza in 2019–2020 reported median ages of 3.9 years for influenza and 8.3 years for COVID-19 [3]. Similarly, a study in Mexico comparing children with COVID-19 in 2020 to those with influenza in 2013–2018 found median ages of 3.7 years for influenza and 5.3 years for COVID-19 [4]. However, these studies were conducted before 2021. More recent data from Poland (2022–2023) showed that children older than 24 months had a higher risk of contracting influenza than COVID-19, a trend similar to our findings [7]. Furthermore, a study from Korea (2015–2022) revealed that infants aged 1 to 11 months were most affected by COVID-19, while influenza infections were more common among children aged 3 to 5 years [6]. These findings demonstrate that the study area and period significantly influence the age epidemiology of children infected with COVID-19 or influenza.

A previous study on adults found a lower percentage of patients with a high fever (≥ 39.0 °C) in the COVID-19 group compared to those with community-acquired pneumonia (CAP) caused by the influenza virus [42,43]. Similarly, a study in China (2022–2023) reported that patients with influenza A had a higher incidence of high fever (≥39.0 °C) than those with SARS-CoV-2 Omicron [8]. In our study, we found that children admitted with a fever exceeding 40 °C in 2024 were more likely to have influenza A than influenza B or COVID-19. Specifically, 40% of influenza A cases presented with a high fever (>40 °C), compared to 3.3% for influenza B and 9.1% for COVID-19 (both *p* < 0.001).

Differentiating children with COVID-19 from those with influenza based solely on symptoms is challenging, as the three most common symptoms in our study—fever (>94%), cough (>82%), and rhinorrhea (>47%)—were prevalent across all periods. A study at CGMH in Taiwan reported similar findings among children visiting the pediatric emergency department (PED) from April to July 2022, with fever, respiratory symptoms (62.9%), gastrointestinal symptoms (23.1%), headache (3.4%), and myalgia (1.6%) being the most common presentations [17]. A 2020 meta-analysis of 37 studies found that children with COVID-19 had similar primary symptoms—fever (48.5%), cough (40.6%), and rhinorrhea (11%)—but with lower prevalence due to variations in viral strains and the inclusion of non-hospitalized children [44]. Children with influenza were more likely to have a cough compared to those with COVID-19, as shown in an adult study [43]. A study in Turkey (2017–2022) reported that fever, cough, and runny nose were more common in patients with influenza, whereas abdominal pain and rash were more frequently observed in patients with COVID-19 (*p* < 0.05) [9]. Additionally, a meta-analysis of studies published between 2000 and 2020 found that sore throat and rhinorrhea were less frequent in COVID-19 cases (11.5% and 9.5%, respectively) compared to influenza A (49% and 44.5%, respectively) and influenza B (38% and 49%, respectively), a trend consistent with our study (Table 2) [45]. Another meta-analysis reviewing literature from 1964 to 2022, as well as a separate study from 2020 to 2021, found that COVID-19 was associated with significantly lower rates of clinical symptoms and abnormal laboratory findings compared to influenza in pediatric patients, a pattern that aligns with our results (Table 2 and Table 3) [10,11]. Similarly, a study conducted in Wuhan, China (2019–2020) reported that the clinical manifestations and laboratory test abnormalities in children with COVID-19 were milder than those observed in children under 5 years old with influenza A [12].

Children with influenza A in 2015–2016 were more likely to experience body aches (45.2%) compared to those with influenza A in 2024, influenza B in both periods, or COVID-19 (all < 26%, *p* < 0.028). Additionally, children with influenza A or B in both periods had a higher percentage of body aches (all > 16%) compared to those with COVID-19 during the two 2022 waves (both 1.3%, *p* < 0.007). Myalgia was more prevalent during the 2009 influenza pandemic (30.12%) than in COVID-19 patients in 2020 (18.97%), as reported in a previous meta-analysis [46].

A 2021 study in Turkey compared laboratory findings between children diagnosed with COVID-19 and those with influenza. The researchers analyzed data from 71 children with COVID-19 and 74 with influenza, all diagnosed before 2021. The study found no significant differences between the two groups in several laboratory parameters, including WBC counts, neutrophil counts, lymphocyte counts, incidence of neutropenia, hemoglobin levels, platelet counts, CRP, AST, or ALT levels [5]. However, lymphopenia was more common in children with influenza (64.9%) than in those with COVID-19 (45.1%, *p* = 0.017). In contrast, our study found that children with COVID-19 in 2024 had a higher percentage of leukocytosis (WBC > 12,000/µL) compared to those with COVID-19 during the 2022 waves (*p* < 0.007) and those with influenza A or B in 2024 or 2015–2016 (*p* ≤ 0.004). A 2020 study in China found that adults with COVID-19 had a lower frequency of leukocytosis, neutrophilia, and lymphocytopenia but a higher likelihood of elevated creatine kinase compared to adults with influenza [47]. That study also showed that leukocytosis (>9.5 × 10^9^/L) was more common in adults with influenza (30.4%) than in those with COVID-19 (16.1%) [42,47]. Another study reported elevated WBC in 75% of adults with COVID-19 compared to 26.83% of those with influenza pneumonia (*p* < 0.01), while neutrophilia (>75%) was more common in adults with influenza (50.4%) than in those with COVID-19 (32.2%) [42,48]. A study in Turkey comparing children with influenza (2017–2018) and those with COVID-19 (2020) found that leukopenia, lymphopenia, and thrombocytopenia were more common in influenza patients than in COVID-19 patients. This aligns with our findings, except for thrombocytopenia, which was not prominent in our study [13]. Similarly, a study in China reported that the influenza A group had significantly lower lymphocyte counts than the COVID-19 group (*p* < 0.001) [8]. A meta-analysis of studies published between 2019 and 2022 found no significant difference in CRP levels between children with influenza and those with COVID-19 [15]. However, after distinguishing between influenza A and B, we observed that children with influenza A in 2024 and 2015–2016 had higher rates of CRP > 1 mg/dL or CRP > 3 mg/dL compared to those with influenza B in 2015–2016 or COVID-19. Our results were consistent with findings from nearly all studies in a meta-analysis, which reported significantly lower CRP levels in COVID-19 patients than in influenza patients [8]. Furthermore, a 2019–2020 study in Turkey identified increased CRP (OR: 7.650; *p* = 0.002) as a key predictor of influenza diagnosis compared to COVID-19 [14].

A Chinese study (2018–2020) found that adults with COVID-19 had lower WBC and neutrophil counts than those with influenza, though both remained within normal limits. Conversely, ALT, AST, creatinine, lymphocyte percentage, and hemoglobin levels were higher in COVID-19 patients, with no significant differences in lymphocyte counts, neutrophil percentages, or platelet counts between the groups [49].

The increased rate of pneumonia in children with COVID-19, influenza A, and influenza B in 2024 was reported for the first time globally. A 2020 study in China found that COVID-19 patients exhibited more frequent imaging features—such as consolidation, crazy paving patterns, rounded opacities, air bronchograms, tree-in-bud signs, interlobular septal thickening, and bronchiolar wall thickening—compared to influenza patients (*p* < 0.05) [47]. Ground-glass opacities were also more commonly observed in adults with COVID-19 than in those with influenza across multiple studies [42,50,51,52,53]. A meta-analysis of studies published between 2000 and 2020 found that the majority of COVID-19 patients had abnormal chest radiology findings (84%, *p* < 0.001) compared to those with influenza A (57%, *p* < 0.001) and influenza B (33%, *p* < 0.001), a trend also observed in our 2024 data (Table 3) [45]. Similarly, a study conducted in Wuhan, China (2019–2020), reported that ground-glass opacities in chest computed tomography (CT) were more commonly seen in COVID-19 patients [12]. COVID-19 appears to have a higher potential for respiratory pathogenicity, leading to more respiratory complications [54]. In line with this, our study found that children with COVID-19 in 2024 had a higher rate of pneumonia (27.3%) compared to those with influenza A (14.8%) or influenza B (16.7%) in 2024.

The strengths of this study lie in its comprehensive design, comparing children with COVID-19 during the first two pandemic waves and in 2024, as well as children with influenza A or B in 2015–2016 and 2024. A limitation of this study is that the data were obtained from a single hospital, which requires validation across diverse populations.

## 5. Conclusions

In conclusion, children with influenza A in 2024 showed a higher incidence of high fever (>40 °C), fever lasting ≥3 days (both >75%), lymphocytopenia (<1000/μL), and CRP > 1 mg/dL. Children with influenza B had a higher incidence of fever lasting ≥3 days (>75%), while those with COVID-19 exhibited higher rates of pneumonia and leukocytosis. Over 43% of children with COVID-19 in 2024 (and during both 2022 periods) were under 3 years old, whereas more than 46% of children with influenza A or B in 2024, or influenza A in 2015–2016, were aged 6–11 years. This study highlights differences between COVID-19 and influenza in children, offering valuable insights for clinicians managing the co-circulation of these viruses. The elevated pneumonia rates in children with COVID-19, influenza A, and influenza B in 2024 compared to previous findings highlight the need for heightened vigilance and prompt management of concurrent pneumonia.

## Figures and Tables

**Table 1:** Gender and age distribution of children with influenza A, influenza B, and COVID-19 infections.

Characteristics	Influenza A		Influenza B		COVID-19			*p* Value
Year	2015–2016 (*n* = 124)	2024 (*n* = 115)	2015–2016 (*n* = 83)	2024 (*n* = 30)	April to July 2022 (*n* = 153)	August to December 2022 (*n* = 76)	January to March 2024 (*n* = 33)	
Male gender, n (%)	37 (29.8)	47 (40.9)	42 (50.6)	18 (60)	67 (43.8)	42 (55.3)	23 (69.7)	<0.001
Age	9 (6–12)	9 (5–11)	11 (4–13)	10 (6–12)	3 (0–8)	2 (1–9)	3 (1–5)	<0.001
Age <1 years	1 (0.8)	1 (0.9)	0 (0)	1 (6.7)	40 (26.1)	15 (19.7)	8 (24.2)	<0.001
Age <3 years	6 (4.8)	5 (4.3)	5 (6.0)	6 (20)	67 (43.8)	38 (50.0)	16 (48.5)	<0.001
Age 3–5 years	31 (20.2)	26 (22.6)	25 (30.1)	0 (0)	27 (17.6)	11 (14.5)	9 (27.3)	0.008
Age 6–11 years	63 (50.8)	57 (49.6)	26 (31.3)	14 (46.7)	46 (30.1)	15 (19.7)	5 (15.2)	<0.001
Age 12–18 years	30 (24.2)	27 (23.5)	28 (33.7)	10 (33.3)	13 (8.5)	12 (15.8)	3 (9.1)	<0.001

Data are shown as number (%) or median (25th–75th percentile).

**Table 2:** Symptoms and signs of children with influenza A, B, and COVID-19 infections.

Characteristics	Influenza A		Influenza B		COVID-19			*p* Value
Year	2015–2016 (*n* = 124)	2024 (*n* = 115)	2015–2016 (*n* = 83)	2024 (*n* = 30)	April to July 2022 (*n* = 153)	August to December 2022 (*n* = 76)	January to March 2024 (*n* = 33)	
Fever	124 (100)	115 (100)	83 (100)	30 (100)	146 (95.4)	72 (94.8)	33 (100)	0.003
Highest temperature > 40 °C	25 (20.2)	46 (40)	12 (14.5)	1 (3.3)	2 (1.3)	4 (5.3)	3 (9.1)	<0.001
Highest temperature > 39.4 °C	68 (54.8)	93 (80.8)	32 (38.6)	16 (53.3)	40 (26.1)	30 (39.5)	10 (30.3)	<0.001
Duration of fever ≥ 3 d	94 (75.8)	87 (75.7)	50 (60.2)	23 (76.7)	47 (30.7)	42 (55.3)	13 (39.4)	<0.001
Duration of fever ≥ 4 d	74 (59.7)	52 (45.2)	29 (34.9)	20 (66.7)	20 (13.1)	15 (19.7)	6 (18.2)	<0.001
Duration of fever ≥ 5 d	37 (29.8)	29 (25.2)	25 (30.1)	11 (36.7)	7 (4.6)	4 (5.3)	0 (0)	<0.001
Cough	124 (100)	115 (100)	79 (95.2)	28 (93.3)	126 (82.4)	65 (85.5)	30 (90.9)	<0.001
Rhinorrhea	112 (90.3)	93 (80.8)	78 (94.0)	25 (83.3)	73 (47.7)	53 (69.7)	22 (66.7)	<0.001
Sore throat	25 (20.2)	41 (35.7)	33 (39.8)	2 (6.7)	13 (8.5)	20 (26.3)	3 (9.1)	<0.001
Vomiting	7 (5.6)	12 (10.4)	5 (6.0)	3 (10)	33 (21.6)	12 (15.8)	3 (9.1)	0.001
Diarrhea	6 (4.8)	6 (5.2)	1 (1.2)	0 (0)	20 (13.1)	11 (14.5)	2 (6.1)	0.001
Headache	25 (20.2)	18 (15.7)	9 (10.8)	3 (10)	13 (8.5)	13 (17.1)	0 (0)	0.017
Sore body	56 (45.2)	29 (25.2)	18 (21.7)	5 (16.7)	2 (1.3)	1 (1.3)	3 (9.1)	<0.001

Data are shown as number (%).

**Table 3:** Laboratory and radiological findings in children with influenza A, B, and COVID-19 infections.

Characteristics	Influenza A		Influenza B		COVID-19			*p* Value
Year	2015–2016 (*n* = 124)	2024 (*n* = 115)	2015–2016 (*n* = 83)	2024 (*n* = 30)	April to July 2022 (*n* = 153)	August to December 2022 (*n* = 76)	January to March 2024 (*n* = 33)	
WBC > 12,000/microliters	1 (0.8)	14 (12.2)	5 (6.0)	0 (0)	2 (2.0)	8 (10.5)	11 (33.3)	<0.001
WBC < 4000/microliters	12 (9.7)	6 (5.2)	17 (20.5)	3 (10)	14 (9.2)	4 (5.3)	1 (3.0)	0.007
WBC < 3000/microliters	7 (5.6)	2 (1.73)	9 (10.8)	0 (0)	7 (4.6)	3 (3.9)	1 (3.0)	0.082
Neutrophilia Neutrophil > 7500/microliters	31 (25.0)	17 (4.8)	12 (14.5)	5 (16.7)	24 (15.7)	11 (14.5)	8 (24.2)	0.253
Neutropenia Neutrophil < 1500/μL	0 (0)	6 (5.2)	0 (0)	2 (6.7)	8 (5.2)	11 (14.5)	0 (0)	<0.001
Lymphocytopenia Lymphocyte < 2000/μL	118 (95.2)	104 (90.4)	62 (74.7)	24 (80.0)	113 (73.9)	42 (55.3)	14 (42.4)	<0.001
Lymphocytopenia Lymphocyte < 1500/μL	87 (70.2)	98 (85.2)	54 (65.1)	23 (76.7)	82 (53.6)	35 (46.1)	8 (24.2)	<0.001
Lymphocytopenia Lymphocyte < 1000/μL	31 (25.0)	64 (55.7)	12 (14.5)	17 (56.7)	81 (52.9)	19 (25.0)	7 (21.2)	<0.001
Anemia by WHO hemoglobin cut-offs	6 (4.8)	17 (14.8)	5 (6.0)	2 (6.7)	24 (15.7)	4 (5.3)	3 (9.1)	<0.001
Platelet < 200,000/μl	31 (25)	11 (9.6)	34 (41.0)	7 (23.3)	41 (26.8)	15 (19.7)	2 (6.1)	<0.001
CRP > 1 mg/dL	63 (50.8)	58 (50.4)	25 (30.1)	11 (36.7)	24 (15.7)	23 (30.3)	9 (32.5)	<0.001
CRP > 3 mg/dL	18 (14.5)	17 (14.8)	4 (4.8)	3 (10)	1 (0.7)	4 (5.3)	2 (6.1)	<0.001
AST > 38 U/L	0 (0)	5 (4.3)	0 (0)	1 (3.3)	71 (46.4)	34 (44.7)	7 (21.2)	<0.001
ALT > 44 U/L	0 (0)	0 (0)	0 (0)	1 (3.3)	12 (7.8)	11 (14.5)	0 (0)	<0.001
Creatinine > 1.2 mg/dL	0 (0)	0 (0)	0 (0)	0 (0)	0 (0)	0 (0)	0 (0)	−
Radiological and clinical confirmed pneumonia	5 (4.0)	17 (14.8)	3 (3.6)	6 (16.7)	18 (11,8)	4 (5.3)	9 (27.3)	<0.001

Data are shown as number (%).

## Data Availability

The original contributions presented in this study are included in the article. Further inquiries can be directed to the corresponding authors.
